# VOC Emissions from Spruce Strands and Hemp Shive: In Search for a Low Emission Raw Material for Bio-Based Construction Materials

**DOI:** 10.3390/ma12122026

**Published:** 2019-06-24

**Authors:** Tereza Adamová, Jaromír Hradecký, Marek Prajer

**Affiliations:** Faculty of Forestry and Wood Sciences, Czech University of Life Sciences, Kamýcká 129, 165 00 Prague 6, Czech Republic; hradecky@fld.czu.cz (J.H.); marek.prajer@gmail.com (M.P.)

**Keywords:** gas chromatography-mass spectrometry (GC-MS), headspace solid-phase microextraction (HS-SPME), chemical treatment, volatile organic compounds (VOCs)

## Abstract

Volatile organic compounds (VOCs) reduce indoor air quality. They are associated with negative effects on human health and wellbeing. In terms of legislation requirements and consumer pressure, VOCs from engineered wood materials are reduced due to use of water based additives and adhesives in their formulation. Therefore, the main source of VOCs remains the raw material—the wood itself. Alternatives to wood strands, annual plant materials, are tested nowadays due to their advantages: The short cycle; the raw material is sourced naturally and can be produced more sustainably; and faster sequestering atmospheric carbon. The aim of this work was to investigate volatile organic compounds emitted from untreated and chemically treated hemp shive and compare the emission characteristics to soft wood strands. Simple, yet effective chemical treatments, like tartaric acid, citric acid and sodium bicarbonate were used in order to reduce VOC emissions. Gas chromatography-mass spectrometry (GC-MS) combined with headspace solid-phase microextraction (HS-SPME) was used to analyse the volatile compounds emissions. Specific VOCs like acetic acid; Benzaldehyde; hexanal, α-, β-pinenes; limonene and camphene were monitored before and after the treatments. Non-target screening was performed to identify the most responsible compound for differentiation of samples according to their treatments. Comparing untreated samples, spruce strands showed highest amounts of total VOCs, while untreated hemp shive showed the lowest. Further, due to the chemical modification of hemp woody core components, such as hemicelluloses, lignin, and extractives, the key VOCs showed significant changes leading to an increase in the amount of total emissions.

## 1. Introduction

Due to a large amount of building materials based on engineered wood, it is nowadays increasingly used in building constructions and their exposure to indoor air, volatile organic compounds (VOCs) released from wood based materials is recognized as one of the factors to influence indoor air quality [1,2]. As people in the 21st century spend a considerable part of their life-time indoors, in certain conditions, they are predisposed to the sick building syndrome [3]. In that case, inhabitants of poorly ventilated buildings are more prone to suffer from various symptoms like headaches as: Eye, nose or throat irritations; dry coughs; allergy reactions; dry and itching skin; nonspecific hypersensitivity; insomnia; dizziness and nausea or difficulty in concentrating; and tiredness. The intense odors may have a negative psychological influence as well [4]. Increasing concerns of human wellbeing are strictly connected to indoor environmental quality. The selection of building materials plays a key role in its occupants wellbeing, thus there is a demand for a reduction of VOCs, even in eco-materials used in today’s architecture and structural application [5,6]. 

The wooden indoor environment is beneficial to its occupants who suffer less tension and fatigue as VOCs emitted from wood actually can have a positive effect on human health. Wood based panels and materials are often composed of wooden fibers, particles, strands or veneers bonded with several chemical compounds and additives [7,8]. Less harmful chemicals are being used due to environmental and health concerns, and the emissions of VOCs from manufacturing-aid additives, glues, coatings and polymers are being steadily reduced [9]. Furthermore, the possibilities to reduce VOCs released from the natural component of panels, wood, still remains. Attempts to reduce VOCs from wood based materials and structures were made by: Applying coatings containing dispersed nanoparticles with high surface to volume ratio [10]; thermal treatment [11]; manufacturing process modifications [12]; or using buffering capacity of other structural components [13]; or even adding scavengers, such as pozzolan, directly into the medium-density fibreboard (MDF) formulation [14]. 

Naturally, the type and amount of VOCs released from wood or other plant material depend on the plant type, life history, interaction with biotic and abiotic factors, diseases, soil quality, nutrition, irrigation, weather and climate conditions, health of the plant, as well as its life cycle period (e.g., hibernation) at the moment of timber material production [15,16]. Apart from cellulose, hemicelluloses and lignin, wood and other botanical fibers are composed of low molecular weight organic chemicals, extractives [17]. Their content varies from 0.5 to 20 weight (wt.)% in wood [18] and from 1 to 3 wt.% in natural fibers [15]. Provided that degradation of lignin and celluloses during the molding process of wood based structural components is minimized, extractives can be considered as a primary source of VOC emissions [19]. Combining benefits of a fast and straightforward production with presumably low VOC emissions, short life cycle, low density and reasonably good mechanical properties, may set structural bio-based materials based on annual plant fibers as a promising alternative to commonly used engineered wood [20,21].

One such a plant is hemp (*Cannabis sativa*), a fast growing wood-like annual plant, native in Central Asia, close to stinging nettle and belonging to cannabinaceae family. Nowadays, it has been planted in many places around the northern hemisphere. Large amounts of the plant can be harvested at a relatively low cost [22,23]. Hemp shive is a woody core of the hemp stalk with outstanding thermal insulation properties. It is characterized by high porosity, low thermal conductivity and high thermal capacity. It has a microstructure similar to hardwoods with three major layers in the cell wall: Middle lamella, primary cell wall and secondary cell wall [24]. Due to its good physical, chemical and mechanical properties, it has been also used in manufacturing of bio-composites, lightweight and insulating concretes and insulation mats in the construction industry. It ensures better elasticity and higher bearing capacity than solid wood, thus it can easily replace the traditional oriented strand board (OSB)-gypsum-water/air barrier-insulation wall composition [22,25]. Hemp based materials are used either raw (shive or fiber, thermal insulation) or processed (together with glues, polymer matrices or other cohesion improving agents). 

Hemp shive and silica sol based novel building composites were investigated using gas chromatography-mass spectrometry (GC-MS). The yield of total extractives reached 6.23% (dry wt.%) followed by low density and enhanced water resistance of a composite [26]. Hemp shive mixed with slaked lime (calcium hydroxide) provides a building material which is solid, durable, soundproof, mold resistant and highly usable bio-based insulation. To become a building and construction material, hemp shive requires minimum processing. Its use represents a more environmentally-friendly way comparing to traditional building materials based on iron or glass, leading to buildings’ ecological balance improvement throughout their entire life cycle. Furthermore, lime-hemp materials contribute to CO_2_ emissions reduction. CO_2_ is absorbed while hemp is planted as well as during the process when slaked lime is curing into limestone (calcium carbonate) again [22,27,28]. 

Since VOCs presence in indoor air is a case of concern (regarding sick building syndrome), confirmation of VOC emissions from building products is needed [16]. The aim of this study was to determine hemp shive (*Cannabis sativa L*.) VOC emissions before and after simple chemical treatments, and to compare VOC emissions intensity with spruce strands’ (*Picea abies*). 

## 2. Materials and Methods 

### 2.1. Chemicals

Tartaric acid (C_4_H_6_O_6_), citric acid—monohydrate (C_6_H_8_O_7_·H_2_O) and sodium bicarbonate (NaHCO_3_) were purchased from Lach-ner (Neratovice, Czech Republic). A crystalline form of all chemicals was further dissolved in distilled water (Millipore-Elix,-Simplicity) to prepare an aqueous solution of a specific concentration. 

### 2.2. Samples

Spruce strands were sourced from an OSB producer. Prior to processing, raw spruce logs were stored outdoors on the producer’s site, unbarked in the first step and split single-stage way in a disk mill into strands. Afterwards, the spruce strands were dried to 3% moisture content. Spruce strands were sampled on the producer’s site before admixing any adhesives. The size of spruce strands was modified to (3–10) × 2 × 1 mm (length × width × thickness) in the wood processing laboratory, Faculty of Forestry and Wood Sciences—Prague. Hemp shive was purchased from a local supplier of sustainable building materials. It was an industrial hemp grown in the UK and processed exclusively for Lhoist UK that uses it for construction purposes. During hemp processing, fiber and other parts of the plant were removed, where the hemp shive was ready to be used for building purposes. The size of individual shive particles varied among (4–10) × (2–5) × 3 mm. Two months prior to the start of experiment, all samples were stored in the laboratory in containers with a low amount of headspace. 

### 2.3. Chemical Treatment

**Figure 1:**
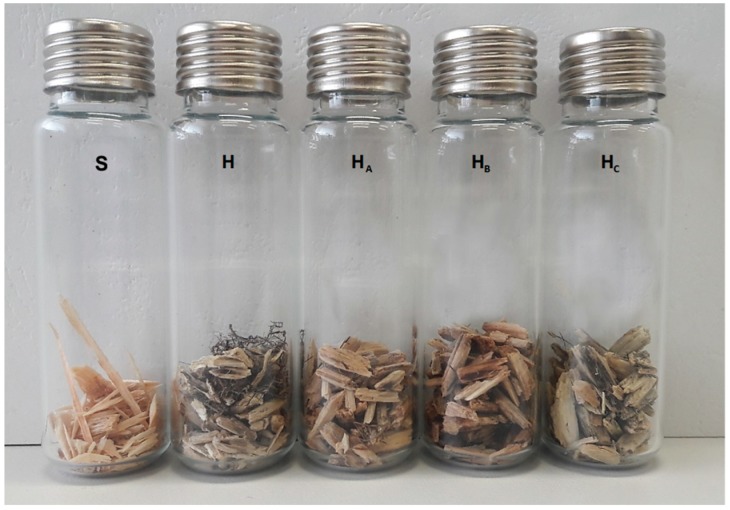
The samples closed in vials ready for GC-MS analysis—visual color change: (S—spruce strands, H—untreated hemp shive, H_A_—hemp shive after tartaric acid treatment, H_B_—hemp shive after citric acid treatment, H_C_—hemp shive after sodium bicarbonate treatment).

Citric acid and tartaric acid, acidic aqueous solutions, previously used by Wilke et al. [29] to reduce VOC emissions from wood-based construction materials, and alkalic sodium bicarbonate [30] aqueous solution were used to modify the hemp shive in order to further reduce its VOC emissions. Low chemical concentrations applied at room temperature were chosen in order not to compromise mechanical properties of the shive. Citric acid, tartaric acid and sodium bicarbonate were dissolved to get 6% (Table 1) in 0.5 L glass beakers, dry hemp shives were added and pressed under the liquid surface using another, smaller beaker. After 24 h of treatment, treated hemp shives were dried in a circulating air oven at 40 °C overnight to a constant water content (10 wt.%) and placed into open headspace vials (each containing a 0.5 g of a sample)—Figure 1. Spruce strands, as well as part of hemp shives, were dried and placed into vials without any chemical pre-treatment—in this study spruce strands served only as a reference. The samples placed in open vials were stored in closed glass desiccators (volume of 24 L) connected to a supply of dry and purified air with a constant flow of 0.5 L·min^−1^ at 23 °C. This approach allowed a controlled air exchange while observing material degradation and volatile emissions [29].

### 2.4. Extraction of Volatiles, GC–MS Analysis and Data Processing 

The samples of un/treated hemp shives and spruce strands were placed separately in desiccators and analyzed for their volatile content after days 1, 3, 7 and 14 using GC-MS. To avoid instrumental sensitivity changes, samples were measured in one sequence. Before analysis, samples were stored airtight closed in vials in deepfreeze (−80 °C). For volatile organic compound collection, solid-phase microextraction, fibers with a divinylbenzen/carboxen/polydimethylsiloxan (DVB/CAR/PDMS 50/30 µm) coating from Supelco (Bellefonte, PA, USA) was employed. Vials were incubated for 10 min to increase volatiles emission from the sample and then, volatiles were collected onto a fibers’ stationary phase for the next 10 min, both at 100 °C.

Gas chromatography coupled to mass spectrometry (GC-MS) was applied for VOCs separation and identification. Basic measurements were performed using Quadrupole Shimadzu GC-MS QP2010 SE—Ultra, applying SLB-5MS capillary column (30 m, 0.25 mm i.d., 0.25 µm film thickness) from Supelco. The injection was performed at 250 °C, while the transfer line was kept at 280 °C. The temperature program was as follows: 40 °C for 1 min and then with grad 5 °C min^−1^ to 250 °C and held for 2 min. Total run time was 45 min. Helium was used as a carrier gas at a flow rate of 1 mL·min^−1^. 

A group of target compounds was based on the literature. Nevertheless, in order not to focus only on few selected compounds, a mass analyzer was operated in SCAN mode (scan speed 2000 ns, range 30–400 *m/z*). The identification of chemical compounds was based on mass spectral similarity with the in-built NIST MS library (NIST, Gaithersburg, MD, USA; 2017 released version). For confirmation of target compounds identity, retention times of respective standards (Sigma-Aldrich; Germany) were used.

Consequently, GC-MS with time of flight (TOF) mass analyzer Pegasus 4D (LECO, St. Joseph, MI, USA) was used to analyze an identical sample set. The aim was to obtain full spectral data for future chemometric evaluation. The sampling procedure remained the same, while for GC separation faster ramping was used. The GC oven temperature program was as follows: 40 °C for 1 min; then ramped at a rate of 10 °C min^−1^ to 70 °C; then at 5 °C min^−1^ to 200 °C and at 20 °C min^−1^ to 28 °C and held for 1 min. The total GC run time was 21 min. 

Automated spectral deconvolution and peak finding algorithms were carried out using ChromaTOF software (LECO, St. Joseph, MI, USA). A build in peak alignment tool, Statistical Compare, was used to align all chromatographic signals with a signal to noise ratio (S/N) higher than 50 in all samples. The data measured using the TOF mass spectrometer were normalized (constant raw sum) and then evaluated using principal component analysis (PCA) and partial least square-discriminant analysis (PLS-DA) in SIMCA 15 software (Sartorius Stedim Data Analytics AB, Malmö, Sweden).

The reported intensities are areas of a unique mass—the specific mass of the compounds’ mass spectrum—that does not co-elute with another compounds signal at a signal’s retention time. For reported compounds RSD (relative standard deviation, expressed as %) from 7 individual measurements was checked and was below 20%.

## 3. Results and Discussion

In this work, hemp shives were subjected to simple chemical treatments, and their VOCs were analyzed and compared to untreated hemp shives and spruce strands. Thirteen key volatile organic compounds including carbonyl compounds, alkanes, aromatic hydrocarbons and terpenes were collected from untreated and chemically treated hemp shives by HS-SPME GC-MS. Target compounds (Table 2) were selected based on a list published in ISO 16000-6 (Annex A) dealing with the building products’ VOC emissions in indoor air [31].

**Figure 2:**
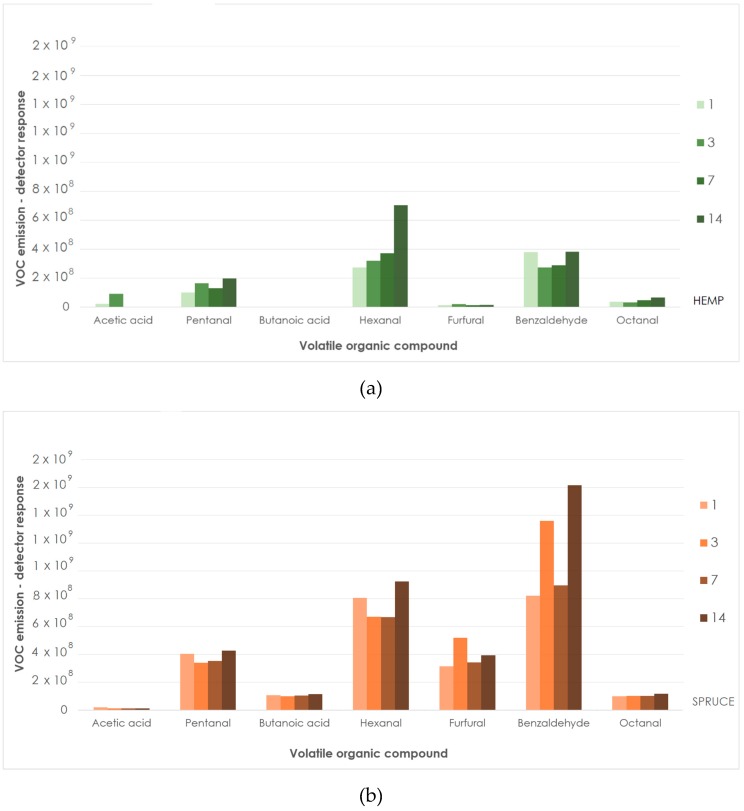
Selected VOC emissions from spruce wood and untreated hemp (day 1, 3, 7, 14; detector response for unique mass) (**a**): untreated hemp shive; (**b**): spruce strands.

In general, a higher number of VOCs, as well as higher detector responses, were observed in spruce strands compared to untreated hemp (Figure 2), especially in case of Pentanal, Hexanal, Furfural and Benzaldehyde. This observation corresponds with an expected higher content of extractives in spruce wood [14,17].

**Figure 3:**
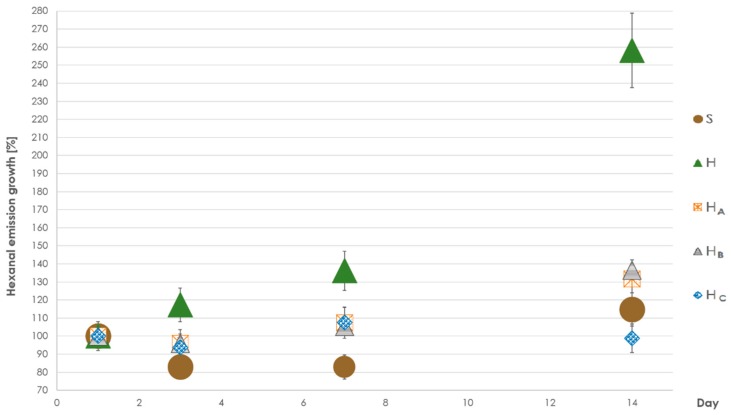
Hexanal emission changes (in %) in tested materials after storage in desiccators (day 1, 3, 7, 14); unique mass intensities first sampling day were considered as 100%. Error bars represent twice relative standard deviation of hexanal intensity measurement in 7 tested samples (8%). (S—spruce strands, H—untreated hemp shive, H_A_—hemp shive after tartaric acid treatment, H_B_—hemp shive after citric acid treatment, H_C_—hemp shive after sodium bicarbonate treatment).

Small test chambers were used to simulate typical interior room conditions where partial changes of air volume were taking place continuously [15]. After 14 days of storage in desiccators with a constant air flow, a slight decrease in intensity signal of above mentioned compounds was observed. A pentanal increase was reported only after sodium bicarbonate treatment and it was later decreasing during storage. Together with more important aldehyde–hexanal, it is known as a product of unsaturated fatty acids oxidation [14]. Both compounds have been identified as causing unpleasant, irritating odors [32] and are known to cause off-flavors in low concentrations in paper [33]. Hexanal, described as grassy [34], kept its initial value after sodium bicarbonate treatment. It was then increasing in all other materials (Figure 3) during storage (especially in case of untreated hemp). 

Both materials in untreated forms emitted similar amounts of acetic acid. Meanwhile acidic treatments enhanced the emission of this compound; sodium bicarbonate treatment led to its inhibition. Unpleasantly smelling butanoic acid was not present in untreated hemp as well as in sodium bicarbonate treated samples (signal below limit of detection—LOD), while the acid treatment significantly increased its abundance. Comparing the untreated and sodium bicarbonate treated hemp shive, aldehyde (hexanal and octanal) emissions lowering effect of sodium bicarbonate was observed, while the emission of other compounds (especially terpenes) rose. 

**Figure 4:**
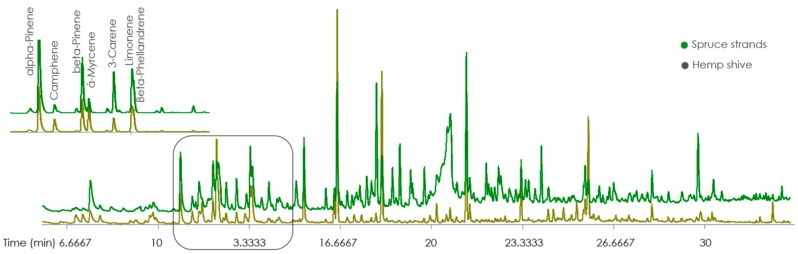
Chromatograms of VOCs extracted from spruce strands and hemp shives (with a focus on terpenes).

A wide variety of terpenes (such as alpha-pinene, beta-pinene, camphene, 3-carene and limonene) was observed (Figure 4) in both samples (spruce and hemp). Nevertheless, it should be noted that even higher responses of terpenes in the case of hemp were still significantly lower than those measured in the spruce samples. Moreover the amount was decreasing with time.

Basic chemometric data evaluation using PCA clearly demonstrated the separation of spruce strands samples from all hemp samples. It was caused by higher relative abundance of characteristic spruce wood volatiles, alpha and beta-pinene. These findings confirmed a difference in released VOCs between untreated spruce strands and untreated hemp shives, showing that untreated hemp shives can be regarded as a safe construction material for indoor air quality. 

**Figure 5:**
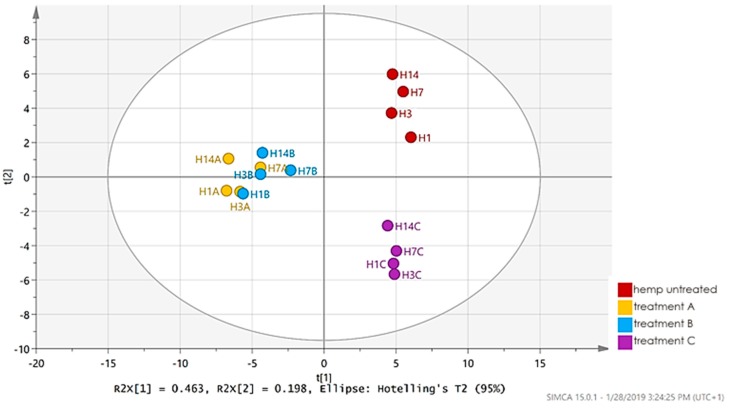
PLS-DA score plot: (H1-14—hemp with no treatment, H1A-14A—hemp shive and tartaric acid; H1B-14B—hemp shive and citric acid; H1C-14C—hemp shive and sodium bicarbonate)

To further investigate differences among hemp treatments, partial least square-discriminant analysis (PLS-DA) was performed. No separation was observed according to the duration of storage, while samples were separated based on chemical solutions treatment (Figure 5). 

**Figure 6:**
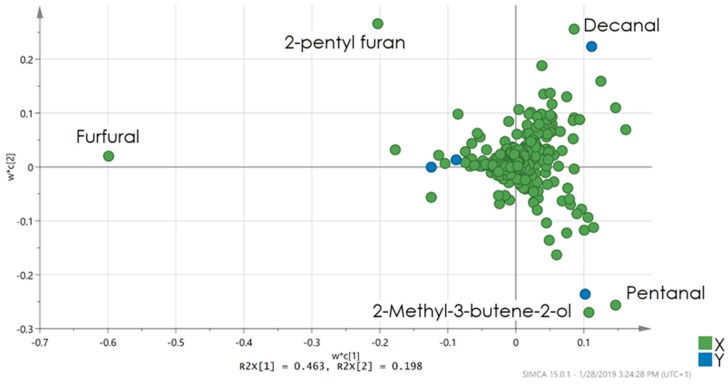
PLS-DA loadings plot presenting significant markers for each treatment. Compounds identification was based on mass spectral similarity and retention index comparison. The identified compounds (pentanal, furfural, 2-pentyl furan, decanal, 2-methyl-3-butene-2-ol) importance is correlating with a distance from the central point; the further the distance, the higher is the markers importance.

In the case of hemp, both acid treated samples formed one group, mainly due to higher abundance of furfural (Figure 6). Furfural may be formed under thermal stress from degradation of polyoses (hemicellulose) [35]. It is often used for industrial manufacturing, food flavoring, for fragrance in personal care products or as a book papers preservative. This compound is considered to be relatively harmless [36]. The samples of sodium bicarbonate treated hemp shive separated due to higher relative abundance of pentanal and 2-methyl-3-butene-2-ol being used as a fragrance ingredient in cosmetics, fine fragrances, shampoos, toilet soaps and other toiletries as well as in non-cosmetic products, such as household cleaners and detergents [37].

Simple chemical treatments did not further decrease VOC emissions from hemp shives. Nevertheless, the amount of released VOCs compounds was still lower compared to untreated spruce strands. 

The chemical composition of raw material used for building materials production represents only one of the factors affecting the quality of indoor air. In addition, the performance of building materials and therefore VOCs release, depends on prevailing thermal and moisture conditions, air pressure difference over the structure, structural design and the quality of construction work, volume of air contained in the indoor space, rate of production or release of the pollutant, the rate of removal of the pollutant from the air via reaction or settling, and the rate of air exchange with the outside atmosphere [20]. The impact of these factors on VOCs release was not considered in presented study. 

## 4. Conclusions

Natural fibers can be used as an alternative to wood in advanced composite applications, in terms of their mechanical properties. To assist in the prediction of the final product influence on indoor air quality, raw solids used for boards processing should be tested to provide information on VOC emissions. Considering raw untreated samples, hemp shive showed a significantly lower amount of total VOCs comparing to spruce. The present analysis did not prove a simple chemical pre-treatment can reduce the overall VOC emissions from hemp. Due to chemical modification of hemp woody core components, such as hemicelluloses, lignin and extractives, the key VOCs showed significant changes, leading to an increase in the amount of total emissions. Nevertheless, particular emissions can be reduced or kept at initial values. In conclusion, material from annual plants like hemp shive proved to be a viable alternative to spruce strands for bio-based materials in terms of VOCs reduction. 

## Figures and Tables

**Table 1:** Spruce strands and hemp shive modifications (S—spruce strands, H—untreated hemp shive, H_A_—hemp shive after tartaric acid treatment, H_B_—hemp shive after citric acid treatment, H_C_—hemp shive after sodium bicarbonate treatment).

SAMPLE/Designation	Spruce Hemp				
	S	H	H_A_	H_B_	H_C_
Treatment	*-*	*-*	*Tartaric acid*	*Citric acid*	*Sodium bicarbonate*
Solution concentration (wt.%)	-	-	6	6	6
Soaking time (h)	-	-	24	24	24

*Note*: all samples were dried up to 10% moisture content prior inserted into vials.

**Table 2:** The intensities of monitored VOC emissions; intensities detector response for unique mass: (S—spruce strands, H—untreated hemp shive, H_A_—hemp shive after tartaric acid treatment, H_B_—hemp shive after citric acid treatment, H_C_—hemp shive after sodium bicarbonate treatment).

	SAMPLES & SAMPLING DAYS																			
COMPOUND	S				H				H_A_				H_B_				H_C_			
	1	3	7	14	1	3	7	14	1	3	7	14	1	3	7	14	1	3	7	14
Acetic acid	20	12	12	1	23	90	2	2	90	62	38	38	177	68	44	35	6	6	3	1
Pentanal	403	340	352	428	100	165	131	198	192	180	149	183	237	214	179	191	1275	1073	615	554
Butanoic acid	105	98	103	113	/	/	/	/	20	20	22	26	22	23	25	26	/	/	/	/
Hexanal	805	669	668	925	273	320	372	704	606	582	650	798	577	554	607	787	379	356	408	375
Furfural	315	520	342	394	12	19	12	16	10638	9191	5609	6672	8427	5639	2557	2419	92	86	59	61
alpha-Pinene	1377	7345	2815	4872	666	285	244	24	697	729	594	279	679	572	639	253	887	782	883	303
Camphene	120	145	63	113	190	83	68	3	224	233	181	89	247	192	202	82	265	236	259	87
Benzaldehyde	821	1360	897	1619	379	274	287	381	791	676	560	467	697	704	558	540	952	873	682	579
beta-Pinene	761	1066	271	2189	441	197	172	/	409	440	391	196	291	286	385	177	649	612	646	233
3-Octanone	/	/	/	3	/	2	2	1	/	/	/	2	3	/	/	/	4	4	3	2
beta-Myrcene	176	212	88	147	381	149	142	7	388	365	312	148	382	293	345	141	525	491	501	164
Octanal	98	100	100	116	36	31	46	65	92	87	81	90	71	76	80	82	41	456	44	51
Limonene	818	896	503	147	475	191	190	89	490	500	500	203	585	426	459	194	602	605	593	214

Note: selected mass areas were divided by 10^6^.
